# Does the severity of untreated dental caries of preschool children influence the oral health-related quality of life?

**DOI:** 10.1186/s12903-023-03274-7

**Published:** 2023-08-10

**Authors:** A Alanzi, F Husain, H Husain, A Hanif, JK Baskaradoss

**Affiliations:** 1 College of Dentistry, Kuwait University, Jabriya, Kuwait; 2 Ministry of Health, Kuwait City, Kuwait

**Keywords:** Caries experience, Untreated dental caries, Oral health-related quality of life, Preschool children, Pufa index

## Abstract

**Aim:**

To assess the impact of untreated dental caries and its severity on the oral health-related quality of life (OHRQoL) of Kuwaiti preschool children and their caregivers.

**Methods:**

Participants were 4- and 5-year-old kindergarten children attending preselected public schools from one of the Governorates in Kuwait. Early childhood caries (ECC) was evaluated by clinical examinations and presented using decayed, missed, filled teeth/surface (dmft/dmfs). The clinical consequences of untreated dental caries were assessed using the pufa (pulp, ulcers, fistula, abscess) index for primary teeth. A structured questionnaire obtained demographic information of children and their caregivers. OHRQoL was assessed using the Arabic version of Early Childhood Oral Health Impact Scale (A-ECOHIS).

**Results:**

Among the 334 participants, 171 were kindergarten level-1 (KG1), and 163 were level-2 (KG2). The overall prevalence of dental caries was 78.9% for KG1 children and 67.4% for KG2 children. Decayed teeth were the main component for both dmft (84%) and dmfs (68%). The total mean (SD) pufa score was 0.54 (1.5), and about 19.2% of participants had at least one tooth with pufa > 0. A total of 207 A-ECOHIS were completed. Both family and child impact scores were significantly higher for children with a dmft score of 1 or more (p < 0.001) or with one or more pufa (p < 0.001). Child impact section scores were significantly higher with the increasing degrees of untreated caries (dt) (p = 0.004).

**Conclusion:**

The severity of untreated dental caries and caries experience had a negative impact on the OHRQoL of Kuwaiti preschool children and their families. Using the pufa index had provided additional information about the effect of the caries severity on the OHRQoL than assessing the caries experience alone.

**Supplementary Information:**

The online version contains supplementary material available at 10.1186/s12903-023-03274-7.

## Introduction

Dental caries is a significant widespread oral disease in children [1]. Early childhood caries (ECC) is defined as “the presence of dental decay in any primary tooth in a child younger than age six years“ [1]. In contrast, severe early childhood caries (S-ECC) is determined when any sign of smooth-surface caries is present in children younger than 3 years of age [1]. Also, from age 3 to 5, S-ECC is considered when one or more cavitated, missing (due to caries) or filled smooth surfaces in primary anterior teeth, or a decayed, missing, or filled surface score of ≥ 4 (at age 3), ≥ 5 (at age 4), ≥ 6 (at age 5)” [1]. Untreated caries could have serious consequences, such as dental pain, difficulty chewing, poor appetite, weight loss, sleep disturbance, and poor school performance [2, 3]. Therefore, untreated caries could influence the children’s oral health-related quality of life (OHRQoL) [4–7].

The standard diagnostic methods used in oral epidemiological studies for assessing dental status are the decayed, missing, and filled teeth index (dmft/DMFT) and ICDAS [8]. Both scoring methods do not give information about the clinical oral consequences of untreated caries [8]. In 2010, an innovative index expressed as pufa/PUFA was proposed in the Philippines to address the advanced stages of untreated dental caries [9]. The index determines the presence of exposed pulp, ulceration of oral mucosa due to remaining roots, a fistula, and an abscess. They have been reported as clinical oral mucosal consequences of untreated caries. The reliability and validity of the pufa/PUFA index have been established [9].

Recent studies in different countries have used the pufa/PUFA index to evaluate the severity of untreated dental caries [10–13]. This index has been recommended to use with the dmft/ DMFT index in populations with a high prevalence of caries to complete the recording of caries experience [8]. Thus, public health decision-makers and oral health care providers can be warned about the necessity of providing adequate oral care services in their communities.

According to FDI World Dental Federation, “Oral health is multifaceted and includes the ability to speak, smile, smell, taste, touch, chew, swallow, and convey a range of emotions through facial expressions with confidence and without pain, discomfort, and disease of the craniofacial complex” [14]. This definition reflects the physiological, social, and psychological attributes essential to the quality of life. Oral disorders have a negative impact on the functional, social, and psychological well-being of children and their families [15, 16]. This has led to a greater focus on the need to assess the OHRQoL along with the clinical assessment of oral health, which would help prioritize care and evaluate the outcomes of treatment strategies. However, the assessment of how children’s oral health status affects their OHRQoL is complex, and measurements of this assessment have only recently been developed.

The prevalence of ECC among Kuwaiti kindergarten children (4 and 5 years old) was extremely high, 68% and 76%, respectively [17], which was close to the prevalence in many developing countries [18]. The rising curve of dental caries experience in young children in Kuwait necessitates a significant emphasis on evaluating its clinical consequences and impact on the oral health-related quality of life (OHRQoL) of young children and their caregivers. Limited attempts have been made to assess the effect of untreated caries on preschool children’s OHRQoL using the pufa index [19–22]. This study aimed to evaluate the impact of untreated dental caries with its clinical consequences and caries experience on the OHRQoL of a representative sample of preschool children and their caregivers.

## Methods

### Study design

This cross-sectional study was conducted in randomly selected kindergarten schools in one major governate (Hawally) in Kuwait. The data collection was intended to be carried out from September 2019 to June 2020. Due to the COVID-19 pandemic, the study ended in February 2020. The ethical approval was obtained from the ethical committee of Health Sciences Centre, Kuwait University (IRB #3495), and the Ministry of Education, Kuwait. Detailed explanatory letters concerning the study’s aims and informed consent were given to the parents of each student through their class teachers. The inclusion criteria included normal healthy cooperative Kuwaiti kindergarten (KG) children (KG-1 = 48–59 months, KG-2 = 60–71 months), proper consent forms signed by parents/guardians, and being present at school on the day of examination. The exclusion criteria were non-cooperative children, children with systemic diseases, and children with enamel anomalies such as hypodontia or amelogenesis imperfecta.

### Sampling and power analysis

According to the most recent data from the Statistics Service System in Kuwait, there were around 4,459 Kuwaiti kindergarten children in the Hawally governorate. The total number of classes is between 110 and 200, with a mean number of students per class: 20 to 30. The sampling design was cluster sampling. The mean and standard deviation of a previous study [23] were used in the sample size estimation. Assuming an effect size of 0.2, Type I error of 5%, and a power of 90%, it was estimated that 255 subjects would be required, which was further rounded off to 300 subjects to compensate for incomplete responses and the design effect. Exploratory method defined by the World Health Organisation (WHO) [24] was employed to determine the number of subjects required to participate from each cluster. It was recommended to select between 20 and 50 subjects from each cluster to assess the prevalence of a disease. Therefore, four schools from the governorate were randomly selected, and from each school, two classes were randomly selected by means of a draw. All the children in each class were invited to participate in the study.

### Subject selection

The predetermined number of schools were randomly selected from the sample frame using computer-generated random numbers. School authorities from each selected school were contacted, and their consent to participate in this survey was obtained. The updated list of classes and the number of students in each class were obtained from each school. The required number of students from each grade was selected from this list. A consent form was sent to the selected students’ parents a week before the school visit, and only those students with a signed consent form from their parents were examined.

### Clinical examination

All clinical examinations were performed by one trained and calibrated pediatric dentist at the school premises (nurse’s room). Examinations were conducted on a school chair, using a portable headlight (Sun-Led Classic, BPR Swiss, Switzerland) and a mouth mirror without an explorer or compressed air. A periodontal probe and gauze were used to remove any debris or excess food covering the teeth.

Dental caries experience was evaluated using the decayed, missing, and filled surfaces (dmfs) and teeth (dmft) indices as illustrated by WHO for epidemiological studies [24]. The caries experience was classified as: caries-free (dmft = 0) and caries-positive (dmft > 0). The severity of ECC was further categorized to: non-severe (dmft = 1–5), and severe (dmft ≥ 6) [25]. The d component which represented the untreated decayed teeth was classified as: (dt = 0), (dt = 1–3), and (dt ≥ 4) [26]. Teeth with white spot lesions were considered healthy [24]. The care index (CI) was also calculated and defined as the number of restored teeth as a fraction of the total number of decayed, missing, and filled teeth (CI = f/dmf × 100).

The pufa index was used to evaluate the clinical consequences of untreated dental caries in primary teeth [8]. Lesions in the surrounding tissues unrelated to a tooth, with detectable pulpal involvement due to caries, were not recorded. The four codes of the pufa index are: ‘p’ when pulpal involvement was present; ‘u’ when there was ulceration; ‘f’ if a fistula was present; and ‘a’ for an abscess. The pufa count was computed per child (p + u + f + a). The count range in the primary dentition was from zero to 20.

Prior to the examinations, the examiner performed a training exercise and calibration for the dmfs/dmft and pufa indices. The training exercise was done using figures from various clinical scenarios. The calibration was carried out with oral examinations of 20 children (not included in the study sample), with a 1-week interval between examinations. The intra-examiner reliability kappa score for the examiner was 0.92.

After the clinical examination, all participant children received incentives (stickers) and fluoride varnish if it was not applied in the last 6 months. Parents/caregivers were provided with data regarding the oral health status of their children. Parents were informed to seek dental services to perform the treatment if the child had carious lesions or complications of the disease.

### Questionnaire

A structured questionnaire was used to gather information from the parent/caregiver. The following information was obtained: demographic information related to the child (child gender, age, and child rank) and demographic information related to the parent (marital status, age of mother, educational level of parents).

The OHRQoL of children was assessed using the Arabic version of Early Childhood Oral Health Impact Scale, A-ECOHIS [27]. The A-ECOHIS consists of 13 questions split into two parts: the child impact section and the family impact section. The child part has nine items related to four areas: symptoms, function, psychology, and child self-image/ social interaction. The family part contains four items related to parental distress and family function.

The A-ECOHIS questionnaire is counted using an easy 6-point Likert scale.The responses we recoded as follows: never = 0, hardly ever = 1, occasionally = 2, often = 3, very often = 4, and don’t know = 5. The total count was calculated by adding the scores of all questions. The response “don’t know” was handled as a missing value, as described in the original study [28]. The scores of the child part ranged from 0 to 36, and the scores of the family part ranged from 0 to 16. Higher scores were specified as presenting more problems and/or a greater impact on OHRQoL [28]. The exclusion criteria included incomplete answers or answers with ‘don’t know’ in two or more items, either child or family domains. The A-ECOHIS and the questionnaire were given to the participating parents/caregivers for completion through their children’s class teacher.

### Statistical analysis

Data were managed and analyzed using the Statistical Package for Social Sciences (SPSS for Windows, version 26.0; SPSS Inc., Chicago, Ill., USA) program. Descriptive statistics (mean, frequencies, percentage) were performed. A Chi-square test was used for nominal or ordinal variables. Data normality was tested using the Kolmogorov-Smirnov test. Cohen’s kappa coefficient (κ) was performed to assess the intra-examiner reliability. To determine the construct validity of the A-ECOHIS instrument Principal Component Analysis (PCA) was performed. PCA analysis was carried out using Bartlett’s test of sphericity and the Kaiser–Meyer–Olkin (KMO) measure. In addition, the internal consistency of the instrument was assessed using Cronbach’s Alpha (⍺). The association between the dependent variable (overall ECOHIS scores) and the independent variables [child factors (sex, age, dmft, pufa) and parental factors (father’s education level, mother’s education level)] was tested using a Poisson regression model. Independent variables that had a p-value ≤ 0.20 in the bivariate analysis were included in the multivariable model. Significant independent variables (p-value < 0.05) were selected for the final model [29]. As ECOHIS score is a count variable prevalence ratio (P.R.) and 95% confidence intervals (CI) were calculated. The level of statistical significance was set at 0.05.

## Results

### Sample characteristics

**Fig. 1:**
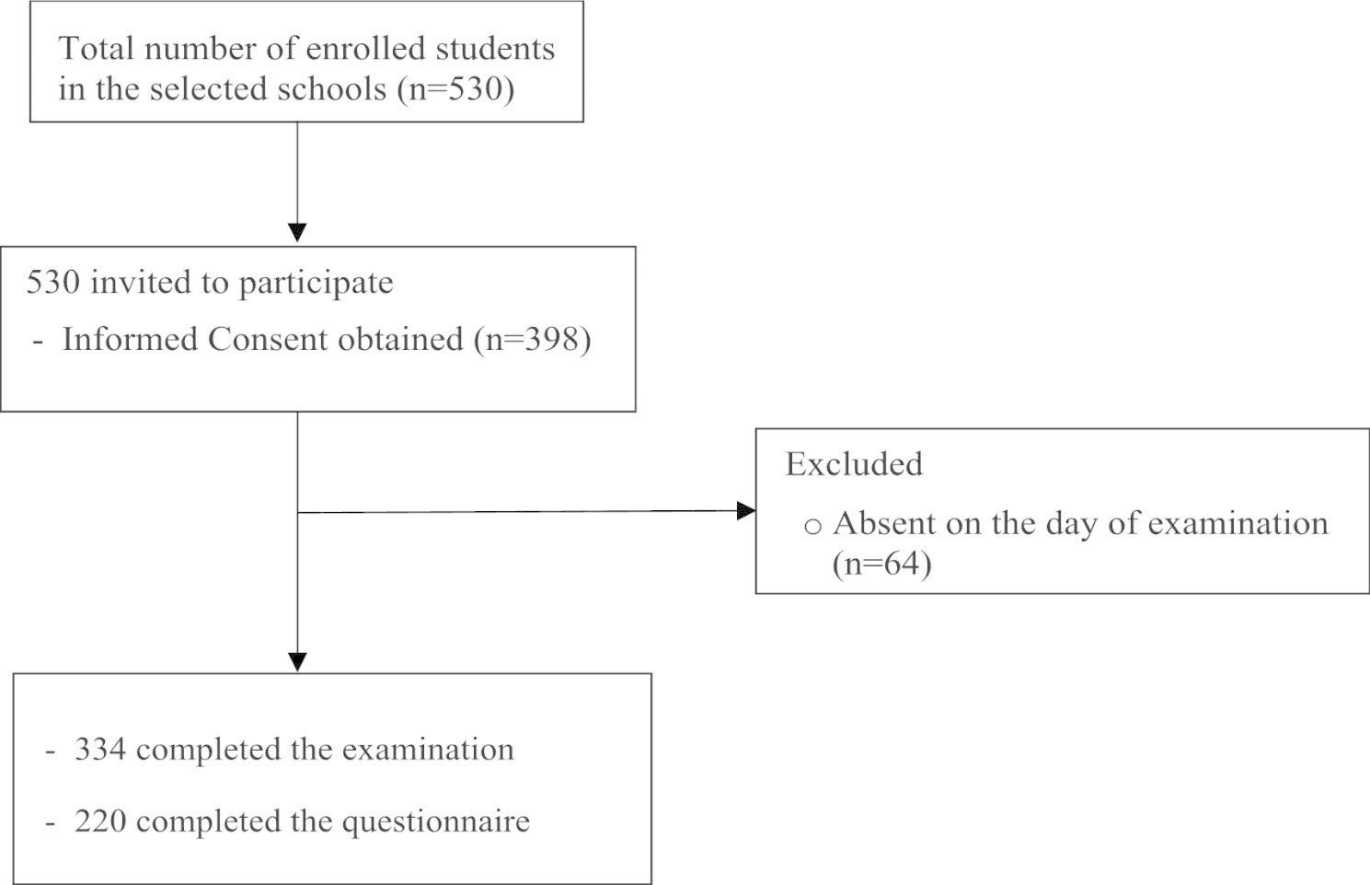
Flow chart of study participants

A total of 530 kindergarten children enrolled in the four selected schools were invited to participate. Written informed consent were obtained from 398 children (75%). Sixty-four children were absent on the day of examination. Therefore, 334 children received the oral examination, with an equal number of participants, boys, and girls (n = 167). A total of 220 questionnaires were returned. Of those, 13 questionnaires were incomplete and excluded (Fig. 1).

Table 1 shows the demographic features of the participant children and their caregivers who completed the A-ECOHIS questionnaire (n = 207). A total of 105 participants were recognized as the oldest children in their families, first-born (25.1%) and second-born (25.6%). In contrast, 32 participants were identified as the youngest children, fifth-born or more (15.5%). Half of the participants (53.1%) had one or two siblings, while 8 participants (3.9%) were the only child in the family. More than two-thirds of children had college-educated fathers (61%) and mothers (76.3%). Most mothers were young (64.3%), aged between 25 and 35 yrs old. Almost all children (91.3%) lived in homes with two married parents. No significant associations were found between the parents’ demographic factors and their children’s caries experience or pufa index.

**Table 1:** Demographic features of the participant children and their caregivers

	Number (n = 207)	Percentage (%)
Child age (months)		
48–59 60–71	109 98	52.7 47.3
Gender		
Male Female	106 101	51.2 48.8
Child rank		
First child Second child Third child Fourth child Fifth and more	52 53 45 25 32	25.1 25.6 21.7 12.1 15.5
Number of siblings		
None One Two Three Four Five and more	8 58 52 41 28 20	3.9 28.0 25.1 19.8 13.5 9.6
Father’s education level		
Less than high school High school College or more	16 64 127	7.7 30.9 61.4
Mother’s education level		
Less than high school High school College or more	7 42 158	3.4 20.3 76.3
Mother’s age		
Less than 25 25–35 yrs old 36–45 yrs old 46 and above	6 133 64 4	2.9 64.3 30.9 1.9
Marital status of parents		
Married Divorced Widow	189 14 4	91.3 6.8 1.9

### Distribution of responses

Among the total 334 participants, 171 were kindergarten level-1 (KG1), and 163 were kindergarten level-2 (KG2). The overall prevalence of dental caries was 78.9% for KG1 children and 67.4% for KG2 children (Table 2). The mean dmft and dmfs scores of all participants were 4.32 ± 4.4 and 9.46 ± 11.1, respectively. The dmft and dmfs were slightly higher in KG2 children, but the difference was not statistically significant (p = 0.182 and p = 0.273, respectively). According to the recorded dmft scores, only a third of all participant children were caries-free (29.6%), the rest either had non-severe (37.8%) or severe (32.6%) caries experience.

**Table 2:** Difference in caries prevalence, dmfs, dmft, white spot lesions, pufa scores among participant children

Variables	Kg1 (48-59 mos)	Kg2 (60-71 mos)	Total
	N = 171(%)	N = 163(%)	N = 334(%)
Caries experience			
Caries-free	56 (32.7)	43 (26.4)	99 (29.6)
Non-severe	62 (36.3)	64 (39.3)	126 (37.8)
Severe	53 (31.0)	56 (34.3)	109 (32.6)
pufa	36 (21.0)	28 (17.2)	64 (19.8)
pulp	33 (19.2)	24 (14.7)	57 (17.1)
ulcer	11 (6.4)	9 (5.5)	20 (6.0)
fistula	3 (1.7)	0	3 (0.9)
abscess	0	1 (0.6)	1 (0.3)
	Mean (SD)	Mean (SD)	Mean (SD)
dmft	4.08 (4.2)	4.56 (4.6)	4.32 (4.4)
dt	3.56 (3.7)	3.69 (4.1)	3.63 (3.8)
mt	0.24 (0.7)	0.40 (0.9)	0.32 (0.8)
ft	0.28 (0.9)	0.48 (1.1)	0.38 (1.0)
dmfs	8.51 (10.1)	10.4 (12.0)	9.46 (11.1)
ds	6.48 (8.4)	6.40 (8.5)	6.44 (8.4)
ms	0.64 (2.5)	1.10 (3.7)	0.87 (3.1)
fs	1.19 (3.9)	2.88 (6.4)	2.02 (5.3)
pufa	0.67 (1.8)	0.40 (1.1)	0.54 (1.5)
pulp	0.44 (1.1)	0.31 (0.9)	0.38 (1.0)
ulcer	0.19 (0.9)	0.07 (0.3)	0.13 (0.7)
fistula	0.02 (0.1)	0	0.01 (0.1)
abscess	0	0.01 (0.1)	0.01 (0.1)

ds = decayed surfaces, ms = missed surfaces, fs = filled surface

dmfs = decayed, missed, filled surfaces

dmft = decayed, missed, filled teeth

Decayed teeth were the main component for both dmft (84%) and dmfs (68%). Dental caries mainly was noticed in both primary maxillary central incisors (31%), followed by the primary mandibular right and left second molars (29.9% and 28.7%, respectively), then the primary mandibular left and right first molars (28.1% and 26.3%, respectively). The primary mandibular incisors were the least affected teeth with caries (< 4%). The most frequently filled tooth was the primary mandibular left first (9.6%) and second molars (9.3%), followed by the primary mandibular right first (8.7%) and second molars (8.1%). The primary maxillary central incisors (5.1%) were the most commonly missing teeth due to caries, followed by the primary mandibular right first molars and left second molars (3.6%, 3.3%, respectively). The most affected surfaces were occlusal, mesial/distal, and labial/buccal. The care index was found to be 6.8%.

The pufa index determined the clinical consequences of untreated dental caries. It was found that 19.2% of all participants had at least one tooth with pufa > 0. The total mean pufa score was 0.54 (± 1.5). The highest pufa count was 16 in one child. Among children with a pufa score > 0, 40.6% had one tooth, 17.8% had two teeth, and the rest had 3–8 teeth. Pulpal involvement (p) was the most frequently scored (17.1%), followed by ulceration (u). The primary maxillary left first molar and mandibular second molars were the teeth mostly affected by pulp involvement. The dmft index was statistically correlated with the pufa index (p < 0.001).

### Overall ECOHIS score

For PCA, Bartlett’s test of sphericity was significant at 0.0001, while the Kaiser–Meyer–Olkin measure was 0.81. According to Scheridan and Lyndall [30], the contents of a tool are considered adequate if the KMO measure value is more than 0.6. The 9 items for the child impact section, the 4 items for the family impact section, and the combined 13 items of the A-ECOHIS had good internal consistency based on Cronbach’s Alpha values (⍺= 0.79, 0.81, and 0.84, respectively).

The frequency of A-ECOHIS responses is listed in Table 3. In the child section, the most frequently reported items were “had difficulty drinking hot or cold beverages” (75.4%) and “avoided smiling or laughing” (75.4%). While in the family section, the most commonly reported item was “had a financial impact on the family”. The ECOHIS scores ranged from 0 to 17 in the child section and 0 to 14 in the family section. About 51.7% of parents/caregivers reported an impact on OHRQoL (ECOHIS score > 0) for at least one ECOHIS item. The mean A-ECOHIS scores were 4.91 ± 4.6 in the child section and 2.16 ± 2.9 in the family section.

**Table 3:** Responses to the Arabic Early Childhood Oral Health Impact Scale (A-ECOHIS) – Children and their caregiver (n = 207)

Impact	ECOHIS response, n (%)					Mean (SD)
	Never	Hardly ever	Occasionally	Often	Very often	
Child impact section						4.91 (4.60)
a. Had pain in the teeth, mouth, or jaws?	84 (40.6)	58 (28.0)	56 (27.1)	6 (2.9)	3 (1.4)	0.97 (0.96)
b. Had difficulty drinking hot or cold beverages	156 (75.4)	31 (15.0)	14 (6.8)	2 (1.0)	1 (0.5)	0.35 (0.69)
c. Had difficulty eating some foods	149 (72.0)	32 (15.5)	22 (10.6)	3 (1.4)	2 (1.0)	0.44 (0.81)
d. difficulty pronouncing any words	139 (67.1)	29 (14.0)	33 (15.9)	4 (1.9)	2 (1.0)	0.56 (0.89)
e. Missed preschool, daycare, or school	122 (58.9)	53 (25.6)	27 (13.0)	5 (2.4)	0	0.59 (0.80)
f. Had trouble sleeping	151 (72.9)	40 (19.3)	15 (7.2)	0	1 (0.5)	0.36 (0.65)
g. Been irritable or frustrated	128 (61.8)	44 (21.3)	31 (15.0)	3 (1.4)	1 (0.5)	0.57 (0.83)
h. Avoided smiling or laughing	156 (75.4)	31 (15.0)	16 (7.7)	3 (1.4)	1 (0.5)	0.37 (0.71)
i. Avoided talking with other children	155 (74.9)	26 (12.6)	21 (10.1)	3 (1.4)	2 (1.0)	0.41 (0.80)
Family impact section						2.16 (2.90)
How often have you or another family member have ………. because of your child’s dental problems or treatment?						
a. Been upset	129 (62.3)	39 (18.8)	26 (12.6)	11 (5.3))	2 (1.0)	0.64 (0.96)
b. Felt guilty	142 (69.1)	24 (11.6)	28 (13.5)	7 (3.4)	5 (2.4)	0.58 (1.00)
c. Had taken time off from work	129 (62.3)	34 (16.4)	34 (16.4)	9 (4.3)	1 (0.5)	0.64 (0.93)
d. Had a financial impact on your family	168 (81.2)	22 (10.6)	12 (6.3)	3 (1.4)	1 (0.5)	0.29 (0.69)

### Caries Severity (pufa) / caries experience (dmft) and ECOHIS score

Table 4 shows the responses to each ECOHIS item in regard to children’s caries experience, untreated caries, and caries severity. As shown in Table 5, The mean ECOHIS scores were 5.16 ± 4.1 among children with non-severe caries experience and 6.48 ± 4.8 among children with severe caries experience compared to 1.79 ± 2.6 among caries-free children. The association was significant (p < 0.001). The family impact score was significantly higher for children with caries experience compared to caries-free children (p < 0.001). The child impact section scores were significantly higher with the increasing degrees of untreated caries (dt) (p = 0.004). The child and family impact sections had significantly higher scores for children with one or more PUFA (p < 0.001).

**Table 4:** Responses to the Arabic Early Childhood Oral Health Impact Scale (A-ECOHIS) in regard to caries experience, untreated caries, and caries severity

Impact	Caries experience (dmft)		Untreated Caries (dt)			Caries Severity (pufa)		
	dmft = 0 (n = 61)	dmft ≥ 1 (n = 146)	dt = 0 (n = 66)	dt = 1–3 (n = 51)	dt ≥ 4 (n = 90)	pufa = 0 (n = 170)	pufa ≥ 1 (n = 37)	
	Mean (SD)	Mean (SD)	Mean (SD)	Mean (SD)	Mean (SD)	Mean (SD)	Mean (SD)	
Child impact section								
Had pain in the teeth, mouth, or jaws?	0.41 (0.6)	1.20 (0.9)	0.44(0.7)	1.02(0.9)	1.33(1.0)	0.88(0.9)	1.41(1.0)	
Had difficulty drinking hot or cold beverages	0.08 (0.2)	0.46 (0.7)	0.11(0.3)	0.39(0.7)	0.56(0.9)	0.32(0.7)	0.62(0.9)	
Had difficulty eating some foods	0.1 (0.3)	0.59 (0.9)	0.23(0.7)	0.51(0.9)	0.66(0.9)	0.41(0.8)	0.84(1.0)	
difficulty pronouncing any words	0.33 (0.7)	0.65 (0.9)	0.39(0.8)	0.75(1.0)	0.63(0.9)	0.55(0.8)	0.76(1.0)	
Missed preschool, daycare, or school	0.31 (0.6)	0.71 (0.8)	0.36(0.7)	0.76(0.8)	0.69(0.9)	0.55(0.8)	0.84(0.9)	
Had trouble sleeping	0.11 (0.3)	0.46 (0.7)	0.15(0.4)	0.53(0.7)	0.43(0.7)	0.32(0.6)	0.59(0.9)	
Been irritable or frustrated	0.16 (0.4)	0.75 (0.9)	0.32(0.7)	0.82(1.0)	0.74()0.8	0.59(0.9)	0.78(0.8)	
Avoided smiling or laughing	0.11 (0.4)	0.47 (0.8)	0.18(0.5)	0.59(1.0)	0.52(0.9)	0.41(0.8)	0.54(0.8)	
Avoided talking with other children	0.16 (0.4)	0.51 (0.8)	0.27(0.7)	0.51(0.9)	0.59(1.0)	0.44(0.8)	0.62(1.0)	
Family impact section								
Been upset	0.20 (0.5)	0.82 (1.0)	0.21(0.6)	0.82(0.9)	0.84(1.0)	0.61(0.9)	0.78(1.1)	
Felt guilty	0.30 (0.8)	0.71 (1.0)	0.27(0.8)	0.69(1.1)	0.76(1.1)	0.54(1.0)	0.78(1.0)	
Had taken time off from work	0.28 (0.7)	0.79 (0.9)	0.27(0.7)	0.90(1.0)	0.77(0.9)	0.61(0.9)	0.78(1.0)	
Had a financial impact on your family	0.08 (0.3)	0.38 (0.7)	0.08(0.3)	0.37(0.7)	0.41(0.8)	0.28(0.7)	0.35(0.7)	

**Table 5:** The association between the difference in caries experience, pufa scores, and A-ECOHIS sections

		Child Impact Section			Family Impact Section		
	N (%)	Mean (SD)	Median (IQR)	P value	Mean (SD)	Median (IQR)	P value
Caries experience							
Caries-free	61 (29.5)	1.79 (2.6)	0 (3.0)	< 0.001^†^	0.84 (1.7)	0.0 (1.0)	< 0.001^†^
Non-severe	81 (39.1)	5.16 (4.1)	4.5 (7.0)		2.18 (2.5)	1.5 (4.0)	
Severe	65 (31.4)	6.48 (4.8)	6.0 (8.0)		3.31 (3.5)	2.0 (6.0)	
Untreated decayed teeth							
None	66 (31.9)	2.45 (3.5)	1.0 (4.0)	0.004^‡^	0.83 (1.60	0.0 (1.0)	0.251^‡^
1–3	51 (24.6)	5.88 (4.4)	5.0 (7.0)		2.78 (3.0)	2.0 (6.0)	
4 or more	90 (43.5)	6.16 (4.7)	6.0 (8.0)		2.78 (3.1)	2.0 (5.0)	
PUFA							
None	170 (82.1)	4.45 (4.3)	3.5 (7.0)	< 0.001^†^	2.04 (2.8)	1.0 (4.0)	< 0.001^†^
1 or more	37 (17.9)	7.00 (5.2)	7.0 (8.5)		2.70 (3.1)	1.0 (5.5)	

^†^Wilcoxon-Mann Whitney test; ^‡^Kruskal Wallis test

PUFA- Pulpal involvement, ulcerations fistula and abscess.

## Discussion

The present study assessed the impact of ECC and its severity on the OHRQoL of a representative sample of Kuwaiti preschool children aged 4–5 years and their families. ECC is recognized as a major public health problem due to its high prevalence and negative health impacts if left untreated [31]. In the current study, the prevalence of ECC was still high among Kuwaiti kindergarten children and similar to what was previously reported in 2010 [17]. The results also show an increase in the severity of ECC which resulted in an increased negative impact on the children’s oral health-related quality of life. The OHRQoL scores were higher, which indicates a poorer OHRQoL among children with caries experience compared to those who were caries-free. The result was in agreement with the published evidence that reported the relationship between caries severity and poor OHRQoL among children of similar age groups [19, 32]. Our sample’s care index value was only 6.8%, indicating a low number of preschool children who received appropriate dental care. The effect of untreated dental caries has been widely reported in several studies conducted on different populations and from various countries [12, 33–35]. This suggests that the relationship between the child’s OHRQoL and ECC is consistent across countries. Also, some systematic reviews [36, 37] concluded that ECC has a negative impact on the OHRQoL of both pre-schoolers and their families. The primary effects of ECC are pain and infection, which negatively alter the eating and sleeping habits of the child [34]. This, in turn, adversely affects the child’s nutritional status, socialization skills, lowered self-esteem, and diminished learning abilities [21]. Zaror et al. reported that severe ECC increased the impact of OHRQoL in pre-schoolers and their families by nearly two times, compared with ECC that is not severe, highlighting the need to focus on the severity of the caries lesions [37]. Nora et al. also concluded that children with a dmft greater than or equal to six presented an even higher impact on OHRQoL [36].

The pufa index was developed by Monse et al. [9] to assess the pulpo-periapical consequences of untreated caries. Clinical consequences (pulp involvement, ulceration, fistula, and abscess) could negatively influence a child’s ability to perform their routine, thereby contributing to poor quality of life. In this study, children with a pufa score of “0” had better OHRQoL than children with a pufa score > 0. This was in agreement with previously published research that reported the impact of the clinical consequences of dental caries on the OHRQoL [21, 22]. This finding suggests that the clinical consequences of the pufa index may have a stronger link with the underlying construct of the OHRQoL. In this study, as with some other articles [9, 32], the ‘p’ score was found to be the major component of pufa, with a proportion of about 70%. Untreated caries could be a reason for toothache which leads to poor performance and a negative impact on the OHRQoL [38, 39]. Knowing the effect of untreated caries based on severity is important, considering the current treatment approach of minimal intervention. In contrast to DMFT/dmft, which provides information only about untreated caries, restorative and treatment status, the pufa index offers vital information on the severity of the carious lesion [9]. Pufa index defines four different clinical stages of advanced carious lesion, which may be more severe than dental caries [9]. The pufa index is easy to use and does not require additional instruments, making it an ideal instrument for use in community or school settings along with the dmft index [9]. Additionally, the presence of pufa in the primary dentition might be one of the most important predictors of caries risk in permanent dentition.

Early Childhood Oral Health Impact Scale (ECOHIS) is one of the most popular measures of assessing the impact of oral health problems on children aged 2–5 years and their families [26]. The ECOHIS consists of 13 items with separate child and family impact sections. The English version of ECOHIS has already been translated and validated in multiple languages [33, 37, 40]. A previous study from Saudi Arabia translated ECOHIS to Arabic (A-ECOHIS) and tested its psychometric properties [27]. In the present study, the mean scores of ECOHIS were higher in the child section when compared to the family. This supports the previously published reports that state parents could tend to underestimate the impact of children’s oral health problems due to their lack of ability or knowledge to identify a child’s social or emotional well-being from their perspective [41]. In a Trinidadian sample, Naidu et al. reported a negative impact of OHRQoL for both the child and family with increasing caries severity [35]. A recent systematic review also confirmed that severe ECC (dmft > 5) showed a greater impact on the OHRQoL in preschool children [37].

In this study, the ECOHIS questionnaire was self-administered, similar to the mode of administration used in the original study by Pahel et al. [26]. Prior to the initiation of the study, it was decided to exclude subjects with ≥ 2 and ≥ 1 “I don’t Know” responses or missing data on the child impact and family impact sections, respectively, as suggested by Jokovic et al. [42]. This resulted in the exclusion of 127 responses. The self-administered mode of ECOHIS may have contributed to the many responses that had to be excluded. Some previous studies have used an interviewer-assisted face-to-face interview for administering ECOHIS [26, 34, 40]. Though Tsakos’s study has demonstrated that the mode of administration does not affect the measure’s performance [43], it may be assumed that using an interview schedule for administering ECOHIS may have reduced the number of excluded cases in this population. A sensitivity analysis was performed comparing the caries experience and severity between children who completed ECOHIS and those who were excluded. No significant difference was noted in the caries prevalence and means of dmft, dmfs, and pufa indices between those 207 children who completed the questionnaires and those who did not (n = 127, P = 0.192, Appendix 1).

Several personal, social, and environmental variables mediate the relationship between oral health status and OHRQoL outcomes. In the current study, the parent’s demographic factors were not associated with the child’s caries experience or OHRQoL. This may be due to the fact that the majority of studied parents were highly educated, and the mothers were young. However, previous studies found that low mothers’ education level has been correlated with a high prevalence of ECC and mean ECOHIS scores in their children [40, 44].

The study’s strengths include using a validated OHRQoL measure, good intra-examiner reliability of the dental examination, acceptable participation rates, and sufficient sample size. However, there were some study limitations. The cross-sectional nature of the current study did not allow assumptions about the directionality of the associations or the explanation of the exposures’ periods. The reliability of the caregivers’ responses was not assessed. Recall bias also possibly influenced the caregivers’ responses. Including only preschool children who live in one governate may have limited the generalizability of the study findings. A well-designed national study using large representative samples from all governates in Kuwait must be conducted to confirm the results reported in this study.

## Conclusion

In summary, untreated early childhood caries and its severity had a negative impact on the OHRQoL of preschool children and their families. Using the pufa index had provided additional information on the influence of caries severity on the OHRQoL than assessing the caries experience alone. Healthcare providers must be aware of the consequences of untreated caries on the overall health and OHRQoL of young children and their families. Understanding this relationship can help develop treatment and preventive protocols for this population.

### Electronic supplementary material

Below is the link to the electronic supplementary material.

Supplementary Material 1

## Data Availability

The datasets generated and/or analyzed during the current study and the full trial protocol are available from the corresponding author on reasonable request.

## List of Abbreviations

ECC: Early childhood caries
DMFT: Decayed missing filled teeth
ECOHIS: Early Childhood Oral Health Impact Scale
KMO: Kaiser–Meyer–Olkin
OHRQoL: Oral health-related quality of life
PCA: Principal component analysis
PUFA: Pulpal involvement, ulceration, fistula, abscess
QoL: Quality of life
SES: Socio economic status
WHO: World health organization
