# Supplementary material for: Does the severity of untreated dental caries of preschool children influence the oral health-related quality of life?
Source: BMC Oral Health. 2023 Aug 10;23:552. doi: 10.1186/s12903-023-03274-7 (PMC10416462; doi:10.1186/s12903-023-03274-7)
Supplement: Supplementary file 1 — Supplementary Material 1 [file 12903_2023_3274_MOESM1_ESM.docx]

**Appendix 1.**

The difference in caries prevalence, dmfs, dmft, white spot lesions, pufa scores

among children who completed the oral exam and ECOHIS vs. those who

completed the exam only

| Variables | | | Exam & ECOHIS Exam only | | | | | | | | | | | | | | | |  | | | | | | | |
| --- | --- | --- | --- | --- | --- | --- | --- | --- | --- | --- | --- | --- | --- | --- | --- | --- | --- | --- | --- | --- | --- | --- | --- | --- | --- | --- |
|  | | | N=207 (%) N=127 (%) | | | | | | | | | | | | | | | | P value* | | | | | | | |
| Caries experience | |  | | | | |  | | | | |  | | | | | | | | | | | | |  |  |
| Caries-free | | 99 (29.6) | | | | | | 37 (29.2) | | | | | | | |  | | | | | | | | |  |  |
| Non-severe | | 126 (37.8) | | | | | | 45 (35.4) | | | | | | | | 0.181 | | | | | | | | | |  |
| Severe | | 109 (32.6) | | | | | | 45 (35.4) | | | | | | | |  | | | | | | | | |  |  |
|  |  | | | | |  | | | | | | | | | |  | | | | | | | | | |  |
| pufa | 37 (17.8) | | | | 27 (21.3) | | | | | | | | | | | 0.185 | | | | | | | | | |  |
| pulp | 31 (14.9) | | | | 26 (20.5) | | | | | | | | | | |  | | | | | | | | | |  |
| ulcer | 14 (6.7) | | | | 6 (4.7) | | | | | | | | | | |  | | | | | | | | |  |  |
| fistula | 3 (1.4) | | | | 0 | | | | | | | | | | |  | | | | | | | | |  |  |
| abscess | 1 (0.4) | | | | 0 | | | | | | | | | | |  | | | | | | | | |  |  |
|  | | | Mean (SD) | | | | | | | Mean (SD) | | | | P value* | | | | | | | | | | | | |
|  | | | |  | | | | | |  | | | | | | |  | | | | |  |  |  |  |  |
| dmft | | 4.34 (4.5) | | | | | | | | 4.28 (4.3) | | | 0.816 | | | | | | | | | | | |  |  |
| dt | | 3.69 (3.8) | | | | | | | | 3.52 (3.9) | | |  | | | | | | | | | | | |  |  |
| mt | | 0.32 (0.8) | | | | | | | | 0.31 (0.8) | | |  | | | | | | | | | | | |  |  |
| ft | | 0.33 (0.9) | | | | | | | | 0.45 (1.1) | | |  | | | | | | | | | | | |  |  |
| dmfs | | 9.04 (11.2) | | | | | | | | 10.1 (11.1) | | | | | 0.526 | | | | | | | |  |  |  |  |
| ds | | 6.03 (8.3) | | | | | | | | 7.1 (8.6) | | |  | | | | | | |  |  |  |  |  |  |  |
| ms | | 0.85 (3.0) | | | | | | | | 0.91 (3.3) | | |  | | | | | | |  |  |  |  |  |  |  |
| fs | | 1.95 (5.3) | | | | | | | | 2.13 (5.3) | | |  | | | | | | |  |  |  |  |  |  |  |
|  | | |  | | | | | | | |  | | | | | | |  |  |  |  |  |  |  |  |  |
| pufa | 0.56 (1.4) | | | | 0.57 (1.7) | | | | | | | | | 0.171 | | | | | | | | |  |  |  |  |
| pulp | 0.44 (1.1) | | | | 0.42 (1.0) | | | | | | | | |  | | | | | | | | | |  |  |  |
| ulcer | 0.19 (0.9) | | | | 0.15 (0.9) | | | | | | | | |  | | | | | | | | | |  |  |  |
| fistula | 0.02 (0.1) | | | | 0 | | | | | | | | |  | | | | | | |  |  |  |  |  |  |
| abscess | 0 | | | | 0 | | | | | | | | |  | | | | | | |  |  |  |  |  |  |
|  | | |  | | | | | |  | | | | |  | | | | | | | | | | | | |

ds = decayed surfaces, ms = missed surfaces, fs = filled surface

dmfs = decayed, missed, filled surfaces

dmft = decayed, missed, filled teeth

* chi-square test for categorical variable, ANOVA for continuous variable
